# A cluster randomized controlled trial of the effectiveness and cost-effectiveness of Intermediate Care Clinics for Diabetes (ICCD): study protocol for a randomized controlled trial

**DOI:** 10.1186/1745-6215-13-164

**Published:** 2012-09-12

**Authors:** Natalie Armstrong, Darrin Baines, Richard Baker, Richard Crossman, Melanie Davies, Ainsley Hardy, Kamlesh Khunti, Sudhesh Kumar, Joseph Paul O’Hare, Neil Raymond, Ponnusamy Saravanan, Nigel Stallard, Ala Szczepura, Andrew Wilson

**Affiliations:** 1 Warwick Medical School, University of Warwick, Coventry, CV4 7AL, UK; 2 Department of Health Sciences, University of Leicester, 24-28 Princess Road West, Leicester, LE1 6TP, UK; 3 University of Leicester and University Hospitals of Leicester NHS Trust, Leicester, Leicester Royal Infirmary, Infirmary Square, LE1 5WW, UK

**Keywords:** Type 2 diabetes, Models of care, Intermediate care clinic, Cardiovascular risk, Multidisciplinary team, General practitioners, Community care, Hospital interface

## Abstract

**Background:**

World-wide healthcare systems are faced with an epidemic of type 2 diabetes. In the United Kingdom, clinical care is primarily provided by general practitioners (GPs) rather than hospital specialists. Intermediate care clinics for diabetes (ICCD) potentially provide a model for supporting GPs in their care of people with poorly controlled type 2 diabetes and in their management of cardiovascular risk factors. This study aims to (1) compare patients with type 2 diabetes registered with practices that have access to an ICCD service with those that have access only to usual hospital care; (2) assess the cost-effectiveness of the intervention; and (3) explore the views and experiences of patients, health professionals and other stakeholders.

**Methods/Design:**

This two-arm cluster randomized controlled trial (with integral economic evaluation and qualitative study) is set in general practices in three UK Primary Care Trusts. Practices are randomized to one of two groups with patients referred to either an ICCD (intervention) or to hospital care (control).

**Intervention group:** GP practices in the intervention arm have the opportunity to refer patients to an ICCD - a multidisciplinary team led by a specialist nurse and a diabetologist. Patients are reviewed and managed in the ICCD for a short period with a goal of improving diabetes and cardiovascular risk factor control and are then referred back to practice.

or

**Control group:** Standard GP care, with referral to secondary care as required, but no access to ICCD.

Participants are adults aged 18 years or older who have type 2 diabetes that is difficult for their GPs to control. The primary outcome is the proportion of participants reaching three risk factor targets: HbA_1c_ (≤7.0%); blood pressure (<140/80); and cholesterol (<4 mmol/l), at the end of the 18-month intervention period. The main secondary outcomes are the proportion of participants reaching individual risk factor targets and the overall 10-year risks for coronary heart disease(CHD) and stroke assessed by the United Kingdom Prospective Diabetes Study (UKPDS) risk engine. Other secondary outcomes include body mass index and waist circumference, use of medication, reported smoking, emotional adjustment, patient satisfaction and views on continuity, costs and health related quality of life. We aimed to randomize 50 practices and recruit 2,555 patients.

**Discussion:**

Forty-nine practices have been randomized, 1,997 patients have been recruited to the trial, and 20 patients have been recruited to the qualitative study. Results will be available late 2012.

**Trial registration:**

[ClinicalTrials.gov: Identifier NCT00945204]

## Background

Configuring services so that all people with type 2 diabetes mellitus (T2DM) can benefit from good quality, target-driven and patient-focused care is a major challenge to healthcare systems such as the UK National Health Service (NHS), especially given the increased and increasing prevalence. In recent years there has been a shift in place of care from hospital to general practice [1]. Although the new contract for general practices set targets for the control of diabetes, concerns were voiced about whether primary care could continue to cope without additional support [2]. Currently, about 70 to 80% of people in the United Kingdom (UK) with T2DM are managed entirely in primary care. There is long-standing and continued evidence of deficiencies in provision of care and significant variations between general practices [3,4]. More recently, differences in quality of care and outcomes have been reported depending on the model of care adopted or staff levels of experience [5,6]. Practices in areas of high deprivation and mix of ethnicity are less likely to achieve adequate levels of control [7-9].

There is evidence from randomized controlled trials that intensive management of T2DM can reduce complications such as retinopathy, nephropathy and neuropathy, as well as reducing the risk of cardiovascular disease [10]. Benefits are seen from improved glycemic control, lower blood pressure and better management of lipids [11-13]. Patients with improved glycemic control also consistently report better functional status and wellbeing [14-16].

It is less clear how benefits seen in individual explanatory trials might be realized in a new integrated service model. A Cochrane review found evidence that structured recall, patient education, and support from specialist nurses can lead to better outcomes [17], and a trial in Danish general practice found that intensive patient-focused management led to significant risk reduction [18]. Economic modeling also suggests that intensive control of risk factors is likely to be cost-effective in the UK [19]. Although the evidence indicates that access to multidisciplinary, community-based services can support general practices in achieving good clinical control of their diabetic patients, and possibly improve patient outcomes, it is unclear what the optimum combination or model might be for service provision.

One method suggested is introduction of intermediate care clinics for diabetes (ICCD). The intermediate care model aims to deliver high-quality care nearer to the patient through multidisciplinary, locality-based teams, with the added opportunity for developing professional expertise in the community and hopefully reducing care costs. A common approach is for medical care to be provided by general practitioners (GPs) with a special interest (GPwSI) in diabetes [20] working with community-based specialist teams. ICCDs began to be introduced in the UK in 2004. A descriptive evaluation found that intermediate care clinics were popular with patients and practitioners and appeared to reduce outpatient attendances by 25% [21]. However, inherent in the ICCD model is a trade-off between the higher levels of technical expertise available in the clinic versus a reduction in the continuity of care as previously provided solely by the GP practice. Continuity of care has been shown to be related to glycemic control [22]. The costs of the two models will also differ, and the cost-effectiveness of ICCD introduction remains unclear.

To date, there have been a number of other ICCD services established in the UK, although the staffing and organization of these varies and none has been introduced under trial conditions with an integral economic evaluation. One service, set up in Cardiff, Wales, has reported some consideration of costs and benefits, although no data on the impact of this ICCD model on costs and patient outcomes are available [23]. Other initiatives include Somerset Community Health [24]; an ICCD service established in Southampton [25]; and an ICCD team set up in Lambeth, London [26]. The latter has reported that the service is marginally cost saving [27]. No other evaluations have been published.

The present study protocol describes a cluster randomized controlled trial of an ICCD intervention set up in three Primary Care Trusts (PCTs) to improve outcomes for primary care patients with T2DM. In accordance with the Medical Research Council’s framework for development and evaluation of complex interventions the trial intervention built on published and ongoing formative research [28,29].

Practices recruited to the study are randomized to either usual care or intervention arm, with the latter having access to the new ICCD clinics. In participating practices, patients with T2DM are invited to take part in the trial. Recruited patients in both arms are given a baseline assessment by a study nurse. This includes measurement of HbA_1c_, body mass index (BMI), waist circumference, blood pressure, urine and lipids. Participating patients are asked to attend a follow-up assessment 18 months after their baseline assessments, when the same measurements are repeated. Questionnaires about quality of life, satisfaction with current services, continuity of care and health service use and personal costs are completed at baseline and follow-up. An integral economic evaluation measures costs in both groups, with a comparative assessment of marginal costs and outcomes. To deal with possible selection bias, risk factor control of all patients with T2DM in intervention and control practices will be compared using anonymised GP data. Additionally, a qualitative study is exploring the views of patients, health professionals and other stakeholders using semi-structured interviews.

The overall aim is to conduct a randomized controlled trial to evaluate the effectiveness and cost-effectiveness of community-based intermediate care clinics in the management of T2DM. Specific objectives are to:

1. Compare the following in patients with T2DM registered with practices that have access to ICCD with those that have access only to usual care:

·control of cardiovascular risk factors including glycemic, blood pressure and lipid control, and cardiovascular risk assessed by UKPDS risk engine;

·quality of life;

·satisfaction with services and continuity of care;

·referral patterns and non attendance rates;

·annual cost per patient with diabetes.

2. Estimate the difference in the cost of the resources used by patients in each arm of the trial, and the cost-effectiveness of the ICCD intervention.

3. Explore the views and experiences of patients, health professionals and other stakeholders.

## Methods/Design

### Study design

**Figure 1:**
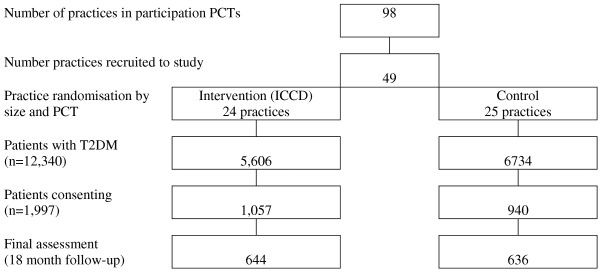
Study design and flow chart.

This pragmatic trial is a two-arm parallel group cluster randomized controlled trial, with randomization at the GP practice level. Practices recruited to the study are randomized to the intervention or control arm. Practices in the intervention arm have access to ICCD. Those randomized to usual care are issued with local guidelines and continue to manage their patients, including hospital referrals, in the usual way. All patients with T2DM in participating practices are eligible for inclusion in the study; up to 100 patients per practice will be recruited. Patients consenting to take part are asked to attend a baseline assessment and then a follow-up assessment at the end of the 18-month intervention period. The trial will assess differences in the percentage of patients achieving adequate control of HbA_1c_, cholesterol and blood pressure (combined) among patients who have access to ICCD in comparison with patients receiving standard care. The study is also designed to examine the effect of access to an ICCD on process measures such as contacts with hospital services and general practice. Additionally, semi-structured interviews are being undertaken in the intervention arm to explore the views and experiences of patients, health professionals and other stakeholders. The study design is shown in Figure 1.

The study protocol was approved by Trent Multi-centre Research Ethics Committee (REC Ref 06/MRE04/41), with amendment to sample size accepted in August 2006.

### Sample size

We initially planned to recruit a total of 5,000 patients from 51 practices (25 in one trial arm, and 26 in the other), with approximately 100 patients being recruited from each practice. Due to losses during follow-up, we expected the final number of patients in each practice to be 72, or approximately 3,700 patients in total.

Estimates for the percentage of patients achieving control in the usual care group are taken from a UK survey [4]. We used HbA_1c_ for our primary sample size calculation as this is the outcome variable for which there is the most robust information on intra-class correlation (ICC). To detect a difference of percentage well-controlled (HbA_1c_ <7.5%) from 50% in the control group to 60% in the intervention group (α = 0.05, Power = 0.8not allowing for clustering, requires a sample size of 408 subjects in each arm. Using an Intra-class correlation (ICC) of 0.047, and with 72 patients in each cluster, the necessary sample size in each arm is 1770, representing a total of 3,540 patients for both arms. This number is also adequate to detect a 10% difference in cholesterol control (from 60% to 70%) and blood pressure control (from 60% to 70%). Estimates of ICC for blood pressure and cholesterol are taken from the UK Asian Diabetes Study (UKADS), a study of care provision for people of South Asian ethnicity with diabetes [30]. Assuming the ICC for our combined primary outcome (adequate control of HbA1C, blood pressure and cholesterol) is 0.05 and achievement is at 20% in the control arm and 30% in the intervention arm, we would need a total of 2,848 patients.

During the recruitment phase, it became clear that we would fall short of the intended 5,000 patients, and of the expected 72 patients per practice. A revised sample size estimate of 40 patients in each practice after follow up (approximately 2,000 patients in total) was proposed and accepted by the ethics panel. Using the same ICC values as previously, and considering the primary outcome, the necessary sample size in each arm is 1,022, for a total of 2,044 (note that this is the necessary sample size for patients retained at follow-up; the sample size for initial recruitment must therefore be larger, or according to our estimates, at least 2,555). This value is approximately sufficient to give power of 80% to detect a 10% difference for both the combined primary outcome and control of blood pressure. We can restore the power to that of the original protocol, before the changes to sample size were made, by considering the detection of a 12% difference for these measures. For control of HbA_1c_ and cholesterol the power is slightly less than 80%.

### Setting/ participants

The study is taking place in general practices in three PCTs in a geographically and socially diverse sub-region of England. Providing good quality care for people with diabetes is a particular challenge for the health services in these areas given the prevalence of the condition, particularly in minority ethnic groups.

#### Practices

All GP practices in the three participating PCTs were first invited to express an interest in participating in the trial. Experience from a similar trial of community cardiology clinics in Leicester suggested that over 80% of practices would agree, as it gives them an even chance of accessing an additional service for their patients [31]. The aim was to recruit 51 practices in total (52% of all eligible practices).

#### Patients

To be eligible for inclusion in the trial, potential participants must be patients in a participating practice, as well as:

a) be adults aged 18 years or older;

b) have T2DM;

c) not have severe cognitive impairment which would preclude active engagement in the trial;

d) not be pregnant at recruitment

e) not have a severe mental illness (for example, anxiety or depression, which would preclude active engagement in the trial);

f) not be receiving terminal care;

g) receive a letter of invitation to the study from their practice;

h) express a wish to participate and provide written consent.

### Study intervention

Following development work, community-based ICCDs were established in the participating PCTs. These are designed to work closely with hospital-based specialist teams and community services, including podiatry and dietetics, in order to support primary care, particularly smaller practices that have the most difficulty achieving good control for their patients. Each ICCD consists of a multidisciplinary team, led by a specialist nurse, with emphasis placed on education and self-management. Medical care is provided by a diabetes specialist or a GP with a special interest (GPwSI) in diabetes and, in practices with access to the service, patients with T2DM are no longer referred to hospital directly. Guidelines for referral to the ICCDs are common across all sites, and include people with poorly controlled T2DM and those with poorly controlled cardiovascular risk factors. Patients are managed by the ICCD team until control of risk factors is achieved and then participants are referred back to primary care. The team members work to local guidelines, adapted from national evidence-based guidelines [32].

At both sites the planned capacity is for 20% of patients with T2DM to attend the ICCD per annum, assuming that on average patients will be seen four times in the ICCD before returning to primary care.

### Outcome measures

The launch of the trial was phased across participating PCTs. Following baseline assessments, practices were randomized and the clinic launched. Eighteen months later follow-up assessments were begun, ensuring that all patients in the intervention arm had 18 months exposure to the ICCD.

#### Primary clinical outcome measure

The primary outcome measure is the proportion of participants reaching all three of the following targets - HbA_1c_ (≤7.0%), blood pressure (<140/80), and total cholesterol (<4 mmol/l), as stated in National Institute of Health and Clinical Excellence (NICE) guidelines for patients with T2DM [33].

If the primary outcome measure is achieved this would represent a significant health gain. The trial aims to detect a 10% difference in the level of control in the intervention arm practices (whether or not patients attend ICCD) compared with practices in the usual care group. Audit data suggest that, in participating practices, about 15% of patients will have this level of control at baseline. We expect an increase of no more than 5% in the control arm.

#### Secondary outcome measures

Secondary outcomes are the proportion of participants reaching targets for individual risk factors (blood pressure, HbA_1c_ or cholesterol) and 10-year risks for CHD and stroke assessed by the UKPDS risk engine [34]. In order to collect sufficient data items to use the UKPDS risk engine, the Practice Record Extraction list was amended to include:

· duration of diabetes (years)

· age at diagnosis (years)

· smoking status at time of diabetes diagnosis

Other biomedical markers include BMI and waist circumference, as well as use of medication, reported smoking, costs, quality of life, emotional adjustment, patient satisfaction and views on continuity.

#### Additional outcome measures

The study also proposes obtaining routinely collected data from all patients with T2DM in participating practices. The following data will be extracted by practice staff and all identifiers will be removed before the data is given to research staff:

1. date of diagnosis of T2DM

2. latest reading with date entered for the following (excluding data entered after the date ICCDs are launched): blood pressure, total cholesterol, HbA_1c,_ and smoking status.

3. socio-demographic data including age (years), sex, and ethnicity index of multiple deprivation score (IMD, derived from patient’s postcode, using IMD 2007).

4. practice code.

Permission to obtain the data will be sought from each participating practice. The data will be extracted by members of staff from the practice using the **M**orbidity **I**nformation **Qu**ery and **E**xport **S**yn**t**ax (MIQUEST) or similar audit tool and will be transferred into an excel file, which will not contain any personally identifiable data. Instructions on how to complete the audit will be supplied by the study team. These data will be collected from the practice by the trial coordinator and will be stored and saved according to Data Protection Act regulations.

#### Patient assessments

Patient assessments are being conducted by a trained researcher on practice premises, using standard operating procedures. Baseline assessments are necessary as GP data are non-standardized and will be up to a year old. It is not feasible for researchers to be blinded to allocation, but outcome measures are all objective; an automated sphygmomanometer is used and results of laboratory blood analyses are extracted from GP or local hospital systems. As assessments take place throughout the day, obtaining fasting blood samples is not feasible.

Patient biomedical markers consist of blood pressure, blood lipids, HbA_1c_, BMI, waist circumference, proteinuria, miroalbumiuria and self-reported smoking. In addition to the European Quality of Life-5 Dimensions (EQ-5D) standardized measure of health status developed by the EuroQol Group [35], health status is also being assessed using the short version of World Health Organization’s Quality of Life. We are also assessing satisfaction with services using the Diabetes Treatment Satisfaction Questionnaire (DTSQ) [36] emotional functioning using the Problem Areas in Diabetes (PAID) scale [37]) and continuity of care using a Continuity of Care Questionnaire [38,39]. All these questionnaires are used at both assessments. Additionally, the baseline assessment includes demographic data and prescribed medication. The GP records of patients are also flagged electronically to encourage reporting of death or change of GP.

GPs will be informed of results contributing to national Quality and Outcomes Framework (QOF) indicators which reward GP practices for the provision of ‘quality care’. These include blood pressure, weight/BMI, HbA_1c_, lipids, microalbuminuria and smoking status. This is done because it is possible that patients attending for a research assessment may be less likely to attend for routine clinical assessments.

#### Economic outcome measures

Data on NHS resource use and personal costs to patients are being collected using standard measures for economic analysis [40]. Direct NHS costs include the number of referrals to and attendances at ICCD in the intervention arm and to hospital in the control arm. We are also recording the number of GP, practice nurse, optometrist, podiatrist and dietician consultations, and total hospital attendances, which include both inpatient and outpatient visits. Data are also being collected on prescribed medication and tests linked to diabetes, including diabetic retinopathy tests. For patients who are referred to ICCD, we note the number of contacts with professionals, as well as any group or educational intervention. Direct personal cost data include personal expenditure such as travel costs and indirect costs such as days off sick from work or reduced hours of employment are being collected. These data are obtained at the time of the follow-up assessment.

Impact on health utility will be assessed using the EQ-5D questionnaire [35,41]. Comparing this with costs (directly and indirectly related to patient management) will enable incremental cost-effectiveness of the intervention to be estimated.

#### Qualitative study

Qualitative data focusing on the views and experiences of patients, health professionals and other stakeholders are being collected. Semi-structured exploratory interviews will be undertaken with a sample of approximately 20 patients who have experienced the intervention. This is planned to be a purposive sample, based on responses to the quantitative questionnaires administered at the end of the study. For patients requiring a caregiver, both the patient and caregiver will be interviewed. Where a patient does not speak English, a translator is engaged. A prompt guide was developed through discussion following a literature review and modified after initial piloting. The interviews focus on the patients’ experiences compared to their previous usual care. Participants are free to choose the day, time and venue for the interview. Wherever possible, interviews will be undertaken face-to-face. If necessary, the interview is undertaken by telephone. The choice of face-to-face or telephone follow-up will be decided by the researcher in consultation with the patient, and is dependent on the patient’s own preference.

In addition, semi-structured interviews with health professionals and other stakeholders are being used to explore views in ICCD and experiences of the service. As with the patient interviews, a prompt guide was developed and modified after initial piloting.

### Patients whose preferred language is not English

We are using translated versions of questionnaires if available (for example the EQ-5D). Questionnaires are only completed by people who are fluent in the language of the questionnaire. In circumstances where a patient’s spoken understanding of a language is better than their understanding of written material, we will ask someone to read the questionnaire to them without attempting any translation. All trial literature (that is, consent forms and Patient Information Sheets) is translated. The consent form and patient information sheet have been translated into Bengali, Punjabi, Gujarati and Urdu languages.

### Trial procedures

#### Practice recruitment

The study team first contacted all practices in the relevant PCTs. Practices were sent an invitation letter and short information sheet via the post, and invited to express an interest in participating in the trial. For practices that express an interest, data on current levels of control of patients with T2DM were examined (% patients with HbA_1c_ <7.5, BP145/85 mmHg or less and cholesterol 5 mmol/l or less). These are routinely collected as part of the GP contract [42] and those practices with the highest proportions of poorly controlled patients were invited to join the study.

Practices are recruited on the understanding that they will have access to results on weight, blood pressure, lipids, HbA_1c_ and microalbuminuria from the assessments of patients belonging to their practice. Blood and urine samples are sent to the laboratory for analysis and results sent to practices in the usual way. Research staff record the patient’s blood pressure and weight in the patient’s electronic health records, subject to prior permission from the practice.

#### Practice randomization

Practices recruited to the study were randomized to an intervention or control arm, stratified by practice size and PCT to achieve similar numbers in each arm of the trial. Practices in the intervention arm have access to ICCD service. The study design anticipated that up to 30% of patients in any practice might be eligible for referral to ICCD. Those practices randomized to usual care are issued with local guidelines and continue to manage their patients (including hospital referrals) in the usual way.

Randomization, stratified by area and GP practice size, was undertaken independently of the trial team by Warwick Clinical Trials Unit.

#### Patient recruitment

All patients with poorly controlled T2DM were approached by a letter from their GP enclosing the study information sheet and reply slip. Once the reply slip was received by the research team, patients were written to or telephoned, as appropriate, to explain the aims of the study and arrange an assessment appointment, at which time written consent was obtained by a researcher. An appointment confirmation letter and further copy of the patient information sheet was sent to the patient by post. The aim was to recruit a total of 2,000 patients from 51 practices, with each recruiting approximately 40 patients with poorly controlled T2DM who would be suitable for referral for ICCD support. In a similar study in Denmark 94% consented [18], but assuming a lower consent rate, as seen in UKADS [30] we expect an average sized participating practice with 150 patients with diabetes to recruit 40 to 100 patients. Those not responding to requests for follow-up attendance were reminded using letters, telephone, email and texting.

To enhance the participation rate, GPs and practice nurses at participating practices have been asked to introduce any eligible patients to the study opportunistically as they consult. The research team also offered evening assessment appointments centrally since evening appointments are not possible at general practices due to earlier closing times. Patient confidentiality is assured at non-practice venues, using a locked secure Case Report Form (CRF) box stored in a secure location. Access to the CRF box is restricted to the trial coordinator and research assessors. Laboratory tests are prepared, sent and received in line with usual hospital protocol. To protect patient confidentiality, the patient’s medical history and medication summary notes are extracted from the practice database by a member of the research team, after informed consent has been obtained from the patient.

### Analysis

#### Statistical analysis

Intention-to-treat (ITT) comparisons of the two groups will be conducted, with the primary dependent variable being the proportion of patients achieving all three clinical targets at 18 months. The main analysis will be based on all patients recruited to the study. As some drop-outs and losses are inevitable, sensitivity style analyses are planned. Missing data imputation will use ‘last observation carried forward’ (LOCF). Secondary analyses will exclude all observations with missing values. Analyses will use a mixed effects logistic regression model, and will adjust for baseline characteristics at both practice and individual level. The former may include practice size and deprivation index, and the latter age, gender, BMI, ethnicity, employment status, educational level and length of time since diagnosis. Characteristics will be included using a forward selection model-building strategy, with only variables significant at the 10% level selected. Odds ratios (OR) will be estimated with 95% confidence intervals, adjusting for potential confounding variables and allowing for the effect of the cluster randomization [43].

Further analysis will examine secondary outcomes (achievement of individual targets for blood pressure, HbA1c and lipids), again adjusting for confounders and allowing for the cluster randomization. In addition, UKPDS 10-year risk scores for CHD and stroke will be analyzed using a linear mixed model.

Tertiary analysis of questionnaire responses will consider longitudinal continuity, flexible continuity, relational continuity and term and boundary continuity following the NCCSDO methodology on ‘continuity of care in type 2 diabetes’ [44]. We will also compare the levels of ICCD use by the intervention practices, and the drop-out rates for all practices.

Finally, all analyses will be re-run with added data from MIQUEST covering all diabetes patients in a practice whether recruited to the trial or not [45]. This will lessen the number of variables available, but otherwise all analyses for primary and secondary endpoints will be repeated.

#### Economic evaluation

Economic evaluation methods will, as far as possible, adhere to the recommendations of the NICE Reference Case [46]. The economic evaluation will consist of a within-trial analysis and economic modeling. Within trial analysis will compare direct costs and 18-month outcomes of patients randomized to intervention versus control. The primary perspective adopted will be that of direct NHS and patient costs. A costing study will record intervention costs (to include staff time, capital, overheads and consumables), other NHS resource use (for example, GP visits, hospital visits, and admissions) and selected patient expenditure. Unit costs for health care resources will be derived from local and national sources and performed in line with best practice [47]. Costs will be standardized to current prices where possible. Because of the short follow-up period, we will not discount costs or benefits.

Comparison will be made between baseline and follow-up to estimate incremental cost-effectiveness ratios (ICERs) comparing the intervention with the control group in terms of the primary outcome measure (patients achieving all three clinical targets) and costs [40]. Quality of life (EQ-5D) over the study period will be used to generate quality-adjusted life-years (QALYs) [48]. Outputs will be presented as ICERs, cost-effectiveness acceptability curves and expected net benefit. Sensitivity analyses will consider key cost drivers and factors that might affect the outcomes measured in order to explore uncertainty in the conclusions drawn.

#### Qualitative study

All interviews are being audio-recorded with the participants’ consent and later transcribed verbatim. Participants are allocated a study number to ensure confidentiality. Interviews will be analyzed using Framework analysis [49], a systematic and comprehensive method of classifying and interpreting qualitative data particularly concerned with generating policy- and practice-orientated findings [50]. The process will begin with familiarization with the data and identification of both broad and sub-themes, followed by continual reassessment and reinterpretation of these emerging themes and the original transcripts. A ‘framework’ for each theme will be created in which data will be charted against individual participants. This process will retain the original context and quotes, and facilitate exploration of the range of comments made under each theme and the relationships between themes.

## Discussion

The present study will provide a robust estimate of the effects of introduction of intermediate care clinics for diabetes to support the care of patients in UK primary care, and the costs associated with any improvements in outcomes produced. The ICCD model aims to deliver high quality care through a multidisciplinary, locality-based team, with the added opportunity for developing professional expertise in the community. The results of this trial will be of direct policy relevance to the development of diabetes services in the UK, and will provide clinical commissioning groups with evidence to inform decisions on commissioning services to improve control in T2DM. Diabetes and service reconfiguration are both national research priorities but there is an urgent need to base policy on evidence. Modeling suggests the ICCDs will be cost-effective in reducing complications and consequent hospital admissions, as well as producing societal benefits such as reduced incapacity to work. If results from this short-term evaluation are positive they will provide an initial evidence base for adoption and for longer-term evaluations to assess the benefits predicted from modeling. The qualitative study will help identify factors influencing service uptake and, if the trial finds no significant effect, why efficacy was not demonstrated. Forty-nine practices have been randomized and 1,997 patients recruited to the trial. Follow up finished in October 2011 and the results will be available in late 2012. It is not possible at present to comment on the effectiveness or cost-effectiveness of an ICCD service.

## Trial status

At the time of submission, patients were being recruited to the qualitative study, and both follow-up assessments and MIQUEST searches were ongoing.

## Abbreviations

BMI, Body mass index; CHD, coronary heart disease; CRF, Case report form; GP, General practitioner; DTSQ, Diabetes Treatment Satisfaction Questionnaire EQ-5D ,European Quality of Life-5 Dimensions; GPwSI, GP with a special interest; ICC , Intra-class correlation; ICCD , Intermediate care clinics for diabetes; ICERs, Incremental cost-effectiveness ratios; IMD , Index of multiple deprivation; ITT , Intention-to-treat; LOCF , Last observation carried forward; MIQUEST , Morbidity Information Query and Export Syntax; NHS , National Health Service; NICE , National Institute of Health and Clinical Excellence; OR , Odds ratio; PCT , Primary Care Trust; PAID , Problem Areas in Diabetes scale; QALYs , Quality-adjusted life-years; QOF , Quality and Outcomes Framework, a system for the performance management and payment of general practitioners in the National Health Service; T2DM , Type 2 diabetes mellitus; UKADS , UK Asian Diabetes Study; UKPDS , United Kingdom Prospective Diabetes Study.

## Competing interests

KK has acted as a consultant and speaker for Novartis, Novo Nordisk, Sanofi-Aventis, Lilly and Merck Sharp & Dohme. He has received grants in support of investigator and investigator-initiated trials from Novartis, Novo Nordisk, Sanofi-Aventis, Lilly, Pfizer, Boehringer Ingelheim and Merck Sharp & Dohme. MJD has received funds for research, honoraria for speaking at meetings and has served on advisory boards for Lilly, Sanofi-Aventis, Merck Sharp & Dohme and Novo Nordisk. The other authors declare that they have no competing interest.

## Authors’ contributions

AW is the Principal Investigator. KK, AW and RB initially conceived the study. AW, KK, RB, SK and JPO designed the study. PS, JPO, KK and MJD additionally oversaw local intervention development and led recruitment and local research nurses AH coordinated the study, including producing trial materials. NR contributed to study design with particular reference to epidemiological and statistical methods/issues. NS also contributed to study design and RC and NS led on statistical analysis. AS led on health economics, and DB led on economic analysis and the writing of this manuscript. NA led the qualitative work. All authors read and approved the final manuscript on behalf of the ICCD Study Group. The views and opinions expressed are those of the authors and do not necessarily reflect those of the NIHR SDO Programme or the Department of Health.
